# Testing the effect of cooperative/competitive priming on the Prisoner’s Dilemma. A replication study

**DOI:** 10.1371/journal.pone.0209263

**Published:** 2018-12-20

**Authors:** Anabel Belaus, Cecilia Reyna, Esteban Freidin

**Affiliations:** 1 Instituto de Investigaciones Psicológicas (IIPsi), Consejo Nacional de Investigaciones Científicas y Técnicas (CONICET) - Universidad Nacional de Córdoba (UNC), Córdoba, Argentina; 2 Instituto de Investigaciones Económicas y Sociales del Sur (IIESS), Consejo Nacional de Investigaciones Científicas y Técnicas (CONICET), Bahía Blanca, Argentina; University of Zaragoza, SPAIN

## Abstract

The replicability crisis in psychology demands direct replications to test the reliability of relevant phenomena. Prime-to-behavior effects have been an area under intense scrutiny given its surprising results. However, intuitive unsurprising effects have been mostly neglected, while they may lack robustness as well. In the present study, we focused on an intuitive prime-to-behavior effect in which Kay and Ross (2003) used a 2x2 design to test cooperation/competition priming crossed with an explicit/non-explicit construal of a Prisoner’s Dilemma (PD). They found a stronger assimilation effect of priming when the situational construal anteceded the decision, but we could not reproduce their findings in the present close replication, despite counting on higher power. Even with limitations due to the unavailability of original materials, this replication presents evidence that questions the existence of the original finding, and highlights the need for further replications to get a deeper understanding of the hypothesized effect. The complete project is available at: https://osf.io/dhfns/.

## Introduction

Prime-to-behavior is a psychological phenomenon in which cognitively activated constructs, such as semantic categories or goals affect subsequent behavior [1]. Since the seminal work of Bargh, Chen, and Burrows [2] about the effect of elderly primes on walking speed, the prime-to-behavior literature has grown exponentially. Several studies have reported effects of subtle primes on a wide variety of behaviors, such as helpfulness [3], aggression and politeness [2, 4], test performance [5 –6], and forgiveness [7], just to name a few. As well, researchers have looked at the influence of primes on social behaviors with economic externalities, such as, the disposition to share money with another participant (e.g., [8–10]), decision strategies in social dilemmas (e.g., [11–16]), and cheating behavior in laboratory games (e.g., [17–19]). Based on this literature, it has become accepted that priming can have strong effects on people’s behavior, and that the activation of social categories and associated traits may automatically influence actions, dispositions, and manners [20–22]. The wide acceptance of the effect of subtle cues on behavior has also been enhanced by the vast literature on framing effects. Numerous studies from diverse fields show that even small changes in the way information is presented play a strong influence on judgments and decisions (e.g., [23–25]).

Despite the cumulative evidence on prime-to-behavior effects, two important concerns have been lately raised in the literature. First, it is the fact that, though direct replications of these phenomena have increased in the past few years, they are still rather uncommon [26–27]. Second and most worrying, many of the direct replications done have failed to reproduce the original findings (e.g., [26, 28, 29–30]). We further discuss these two issues next.

Direct replications are attempts “*to recreate the conditions believed sufficient for obtaining a previously observed finding and [as] the means of establishing reproducibility of a finding with new data*” ([31], pp. 943). In turn, conceptual replications are about testing theory or earlier results with different methods [32]. In the prime-to-behavior field, researchers seem to have favored conceptual over direct replications [33–34], and although the former offer valuable information and could contribute to the understanding of the mechanisms at work, they do not provide information about the reliability of more specific effects [32–33]. A sounding example of this issue is represented by Doyen, Klein, Pichon, and Cleeremans’ [28] failure to replicate the mentioned seminal work of Bargh and collaborators [2], which is one of the most renown effects in the prime-to-behavior literature, with more than 4696 citations (searched in Google Scholar, November 23^rd^ 2018).

The replication studies by Doyen et al. [28], together with other related events in the field of psychology (e.g., the beginning of collaborative efforts to replicate findings [35]), are considered to be the triggers of what has been called the *replication crisis* in psychology [35], characterized by an increasing concern for the differentiation of true and replicable results from those that are not. Many researchers agree that the psychological scientific literature might contain a relevant amount of false positives due to the effect of publishing bias and file drawing (e.g., [36–38]). In consequence, attention has been drawn to the relevance of direct replications to correct this state of affairs (e.g., [26, 33, 29]). Most replication studies have only addressed the reproduction of surprising results though. This makes sense because there are high incentives from editorial teams in top journals to publish attention-calling results, which may create a bias in the direction of publishing surprising false positives, with the corresponding file drawing of non-significant results. Nonetheless, we are here concerned with a neglected area of potential false positives, namely unsurprising findings which may be difficult to detect because of their intuitive appeal. The fact that some of these findings match common-sense views may make it easier for the scientific community to accept them, even without reliable evidence in their support [39].

In this study, we aimed at replicating Kay and Ross´s [11] experiment in which they found an effect of priming cooperation/competition on the construal of and decisions in a Prisoner’s Dilemma (PD) game. The authors of the original study were interested in evaluating the effect of the primes and the mediational influence of a situational construal task (i.e., asking participants to evaluate the situation as cooperative or competitive). They hypothesized that presenting words about cooperation/competition to a person would make her thoughts about cooperativeness/competitiveness more cognitively available. Then, when that person is asked to judge an ambiguous situation such as the PD, she will more likely perceive it as cooperative/competitive. The same would happen when asked to express her beliefs about others´ expected behavior in the situation, or when making her own decision in that context. These authors also proposed that when asked to judge the situation first, people would be more prone to behave in a manner coherent with their previous judgment. That is, the activity of consciously judging the situation and others’ level of cooperativeness/competitiveness would enhance the priming effect on behavior. Indeed, Kay and Ross [11] reported evidence in favor of these hypotheses: participants in the cooperative condition considered cooperative names as more appropriate for naming the abstractly-described PD, expected more cooperation from others, and expressed a higher intention to cooperate themselves than participants in the competitive condition. Also as predicted, they found that the priming effect on the behavioral intention in the PD was stronger when the situation was consciously judged before than after making the decision.

Since their publication, Kay and Ross´ findings have been widely cited (according to Google Scholar, 142 times by November 2018). Nonetheless, it is worth noting that most articles in which this research has been referred to do not present relevant empirical data to determine the direct or conceptual reliability of Kay and Ross´ findings, but mention their results as an example of a strong reliable phenomenon (see e.g., [40–42]). This may seem surprising given that, first, it was a fairly novel finding, and second, it was based on an underpowered experiment, which included only 56 participants in a 2x2 between-subject design. However, the intuitive appeal of the reported effects may have unjustifiable increased researchers´ appraisal of their reliability.

To our best knowledge, there are three conceptual replications of the effect originally reported by Kay and Ross [11], which are reported on the same paper by Kay, Wheeler, and Smeester [12]. In these replications, one of the original authors (Aaron Kay) was involved, and, although every replication is instructive, it is relevant to have in mind that those carried out by the original authors or in the same laboratory might be more prone to a confirmation bias, which is why independent replications are considered more informative [43–44]. In these conceptual replications, Kay et al. [12] reported three studies related to Kay and Ross´ [11]. In the first study, they employed the same priming stimuli used by Kay and Ross [11] and found a statistically significant effect of priming on participants´ judgment of a hypothetical person. In the second and third studies, they used a different task (a subliminal priming task) to prime cooperation/competition and found that the prime influenced the decision in the PD.

In this article, we report two attempts to replicate Kay and Ross study [11]. The closer replication of the original design is presented in the main text, whereas the second replication is presented as an appendix (see S1 File). The replication described in the main text involved reproducing Kay and Ross’ 2x2 factorial design without economic incentives as it was done in the original study. For this experiment, we also relied on a large sample to reach 95% power, and pre-registered the study using the Open Science Framework webpage (osf.io/6uhkb). In the replication reported in the Appendix, we focused solely on the priming effect on the decision in the PD after (but not before) participants made the situation construal tasks. That is, we only reproduced the experimental treatments related to the priming manipulation (cooperation/competition), not those designed to test the effect of the situational construal (explicit/non-explicit construal). Also, in this experiment, we monetarily incentivize participants´ decisions (see S1 File for further details).

## Materials and methods

### Participants

For this experiment, we estimated the sample size needed to achieve a 95% power considering the smallest effect size reported by Kay and Ross (*n*^2^_p_ = .08), and we aimed for a sample size of at least 202 participants (Calculated using G*Power [45]).

The present sample consisted of 346 undergrad students from the National University of Cordoba (age range: 18–35 years old; mean: 21.68; 61% women) who took part in one of 30 sessions, each of which had between 8 and 14 participants. Subjects participated only once and had neither taken part on the preliminary studies described below, nor in similar studies before.

All participants signed a written consent form. Present protocols were reviewed and approved by the Bioethical Committee of the Hospital Municipal “Dr. Leónidas Lucero”, Bahía Blanca, Argentina.

### Materials and design

We asked Kay and Ross for the original protocols and insights about their experiment. Unfortunately, although they showed interest in this project, they replied that the original materials were no longer available, and that they were unable to recall the requested details. Therefore, we used our best judgment to design materials that were not explicitly specified in the original paper.

We did a 2x2 factorial design with priming condition (cooperative vs. competitive priming) and situational construal order (construal first vs. decision first) as fixed factors determining four independent between-subject conditions.

The priming manipulation was performed using the Scrambled-sentence task [46]. This task consisted of 24 non-grammatical five-word sentences that participants had to rearrange into grammatically coherent four-word sentences. Out of the 24 sentences, 16 included words related to the priming treatment, and the remaining sentences were intended to be neutral in terms of their relationship with the concepts of cooperation and competition. A random half of the participants received the cooperative primed sentences, while the other half completed competitive primed sentences. Stroebe and Strack [47] have called attention to the fact that a replication with an exact copy of materials from the original study does not necessarily guarantee a faithful reproduction of the intended variations. In turn, it is fundamental to ensure that manipulations and measures are adequate operationalization of the theoretical construct explored in the original study. Taking into consideration that Kay and Ross’ study was done in English with US participants, we consider essential to evaluate and adjust materials to the language (Spanish) and culture of the population sampled in the present study (Argentine university students).

With the goal of adapting the scrambled-sentences task to the population of students from the National University of Cordoba, Argentina, we conducted three preliminary studies. The first preliminary study was aimed at creating a list of words related to cooperation or competition. Eighty nine students from the National University of Cordoba completed an online survey and listed as many words as possible related to a target word that was either “cooperation” or “competition”. With their responses we calculated the frequency of each reported word, and used such frequency to rank the words separately for the cooperation and competition conditions. In the second preliminary study, 126 participants (undergrad students from National University of Cordoba) indicated for each word from a list of 30 words how close the relationship was to the target word (cooperation or competition; 1 = “very close” to 4 = “very distant”). The list included the words from Kay and Ross’ original study [11] and also the most frequent words from the first preliminary study described before. We built a ranking according to the sum of the “closeness” scores for each word from which we selected the words to include in the Scramble-sentences task. The inclusion criteria prioritized words from the original study, and words perceived to relate to the target words intermediately or highly. In the selection of words, we also took into account the similarity in the mean closeness scores between cooperative and competitive conditions. Last, we conducted a third preliminary study with 18 undergrad students from the National University of Cordoba to compare ratings of difficulty and time to completion of the Scramble-sentence task between the cooperative and the competitive treatments. We observed no significant differences between conditions for the perceived difficulty or completion time. See Supplementary material S2 File for a complete report and analytical data of the preliminary studies.

The tasks used for the situational construal evaluation and the decision in the PD game were translated into Spanish from the text used in the original article. However, given that we did not count with the complete set of original materials, the general instructions were created based on our best judgment.

In terms of the PD-related tasks, first, participants read a neutral description of the PD game, which was followed by the construal task, the beliefs task and the decision task, not necessarily in that order (see the procedure below for more details). The construal task involved rating possible names for the game. Participants had to rate the appropriateness (from 1 = “not appropriate at all” to 9 = “extremely appropriate”) of five names as descriptors of the PD. Two names connoted cooperation (The Community and Group game), two names connoted competition (The Battle and Confrontation game), and one name was neutral (Numbers game). We based the selection of the names on the ranking generated in the second preliminary study described above to ensure that this measure was adequate to the target population. We chose words that had been reported in high frequencies and that were equal or similar to the names used in the original study. Similarly to the original study, the presentation order of the names was counterbalanced among participants in three possible sequences starting either with a competitive name, a cooperative name, or the neutral name. In the beliefs task, participants had to indicate the percentage of people they believed would choose to cooperate and the percentage they believed would choose to defect (the sum had to reach 100%). The presentation order of these two tasks (rating names and expressing beliefs) was also counterbalanced among participants, as it was done in the original study. Last, in the decision task, participants expressed their decision intention in the PD in a scale ranging from. 1 (“I will surely choose A”) to 5 (“I will surely choose B”). As it was done in the original study, the description of the PD was expressed in “points”. Importantly, a random half of participants first responded the name rating and beliefs tasks, and then expressed their behavioral intention in the PD (explicit construal condition), whereas the other half of participants first expressed their behavioral intention and then responded the construal tasks (non-explicit construal condition). Complete protocols are available at OSF (https://osf.io/dhfns/).

### Procedure

Participants were first welcomed and then asked to seat, read and sign the informed consent form in their desks. We made clear that the consent form was the only place where their full names and signatures would be registered. After that, a number was given to each participant, which served to individually identify them without tracking their real identities in order to raffle a monetary prize as a reward for their participation (see details below).

Afterwards, we presented the oral instructions summarizing the general procedure. In the original study, Kay and Ross [11] deceived participants in order to emphasize the difference between the priming task and the PD tasks (i.e., participants were told that they were taking part in two separate studies). However, deceiving participants could have disadvantages and therefore numerous authors discourage its use [48–50]. Hence, we decided to avoid deception but designed the instructions so that the Scrambled-sentences task and the PD tasks were likely perceived as independent. To accomplish this, we orally emphasized to participants that they were taking part in a “Decision Making” study that consisted of *different* tasks. To make the differentiation among tasks more evident, we presented the three tasks as three sets of sheets folded in the middle, which were placed on each desk before participants´ arrival. Each set showed a number on the outside (1, 2, and 3). Participants were instructed to start with Set 1 and to put it inside and opaque envelope after completion, then to read and complete Set 2, and so on (we provided the envelope at the beginning of the study). Set 1 included the Scrambled-sentences task, Set 2 comprised the PD tasks, and Set 3 had three questions to evaluate the comprehension of the PD [51], a funneled debriefing questionnaire [52], and a general socio-demographic questionnaire. Only Set 1 differed between cooperation/competition conditions, whereas Set 2 differed between construal order conditions. Which condition was left in each desk was random and blind to the experimenter. There were no time constrains to complete the tasks, and participants proceeded at their own pace, leaving the room once they had finished responding the questions from Set 3. In their way out, participants received a lottery ticket to participate for two prices of ARS 1000 (approximately US$50) and two prices of ARS 500 to be raffled at the end of the study (i.e., after the last session). Kay and Ross [11] reported giving participants a lottery ticket, but they neither explained, nor were able to specify for us, the amount and characteristics of the prizes used. To our best judgment, we decided to provide monetary prizes in cash, which rewarded participation but were no associated with participants’ PD decisions.

## Results

All the analyses in this section were performed following the analyses design done by Kay and Ross [11]. Also in agreement with the original procedure, we begin by presenting the results from the analyses with the full sample (*N* = 346), followed by a summary of the analysis excluding participants according to two criteria: awareness of the relationship between the priming task and the PD task, and miscomprehension of the PD rules or payoffs.

To begin with, we examined the situational construal measures (i.e., name ratings and beliefs about others’ intention in the PD). The correlation between participants´ ratings of the appropriateness of cooperative names and the correlation between ratings of the appropriateness of competitive names were both significant (*r* = .346, *p* = .000; *r* = .409, *p* = .000, respectively). Based on these significant correlations, we combined the ratings of the two cooperative names together and those of the two competitive names together, obtaining an overall cooperative name rating and an overall competitive name rating, respectively. With these scores, we generated a composite measure of ‘relative appropriateness’ by subtracting the overall competitive score from the overall cooperative score. A higher score in the composite measure indicated a “more cooperative construal of the situation” ([11], p- 683). Contrary to Kay and Ross’ we did not find evidence of a main effect of priming, a main effect of construal order, or a significant priming x construal order interaction for any of the name rating measures. The 2x2 ANOVA for the name rating composite measure showed a non-significant effect of priming (*F*(1, 330) = 0.918, *p* = .339), and a non-significant effect of construal order (*F*(1, 330) = 2.044, *p* = .905; see Table 1), and a non-significant priming x construal order interaction (*F*(1, 330) = 0.014, *p* = .905).

**Table 1:** Priming effects on name appropriateness rating, beliefs about other participants’ behavior, and own behavioral intentions in the Prisoner’s Dilemma game.

	Cooperation condition				Competition condition					Conditions comparison			
	n	*M*	*SD*	95% *CI*	n	*M*	*SD*	95% *CI*	*F*	df	95% *CI*	*p*	Effect size
Rating of Cooperative names	165	10.81	4.31	10.1–11.52	161	9.82	4.94	9.12–10.57	0.3.53	322	-.046, 1.98	.061	*η*^*2*^_*p*_ = .011
Rating of Competitive names	165	8.32	4.59	7.64–9.01	161	8.14	4.32	7.43–8.82	0.164	322	-.773, 1.17	.686	*η*^*2*^_*p*_ = .001
Rating of Neutral name	165	4.94	2.71	4.53–5.35	161	4.97	2.67	4.56–5.4	0.017	322	-.626, .547	.895	*η*^*2*^_*p*_ <.000
Others´ expected cooperation	168	50.94	23.11	46.95–53.94	172	52.9	23.16	49.44–56.34	0.961	336	-2.98, 6.89	.327	*η*^*2*^_*p*_ = .003
Intention to Cooperate	170	2.65	1.41	2.43–2.86	172	2.78	1.44	2.56–2.99	0.707	338	-.173, .433	.401	*η*^*2*^_*p*_ = .002

In turn, a 2x2 ANOVA of beliefs about others’ intentions in the PD revealed a non-significant effect of priming (*F*(1, 336) = 0.961, *p* = .327), a non-significant effect of construal order (*F*(1, 336 (= 2.742, *p* = .099), and a non-significant priming x construal order interaction (*F*(1, 336) = 2.154, *p* = .143).

**Fig 1:**
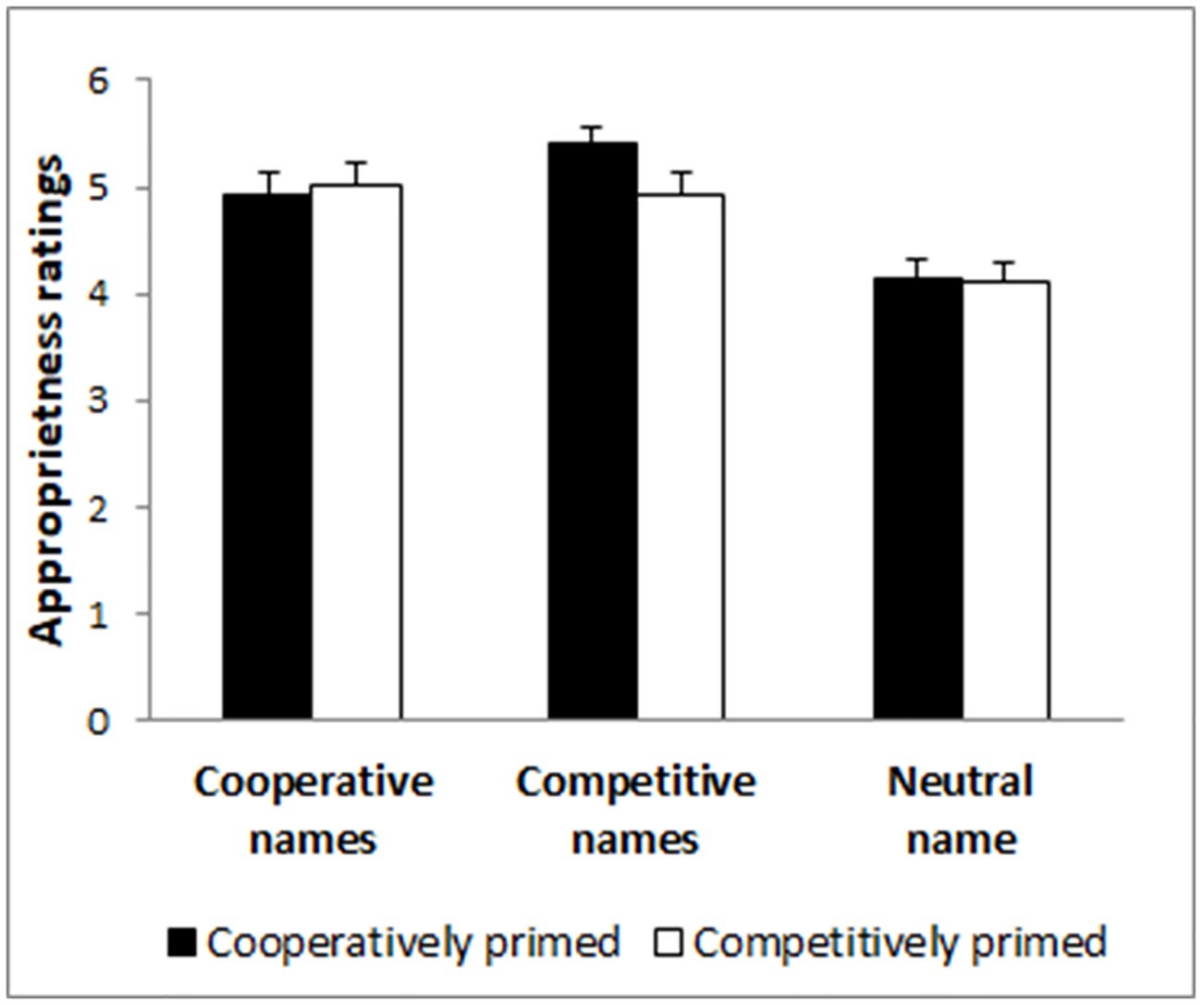
Mean appropriateness ratings for the competitive, cooperative, and neutral names as a function of experimental condition.

In terms of the correlation between the name task composite measure and the beliefs about others’ intentions in the PD, we found a significant positive relationship (*r* = .117, *p* = .035), which may suggest that name ratings and beliefs about others could be linked to an underlying construal of the situation, but that such situational construal was no affected by the cooperative/competitive priming, the construal order, or their interaction (see Table 1 and Fig 1).

**Fig 2:**
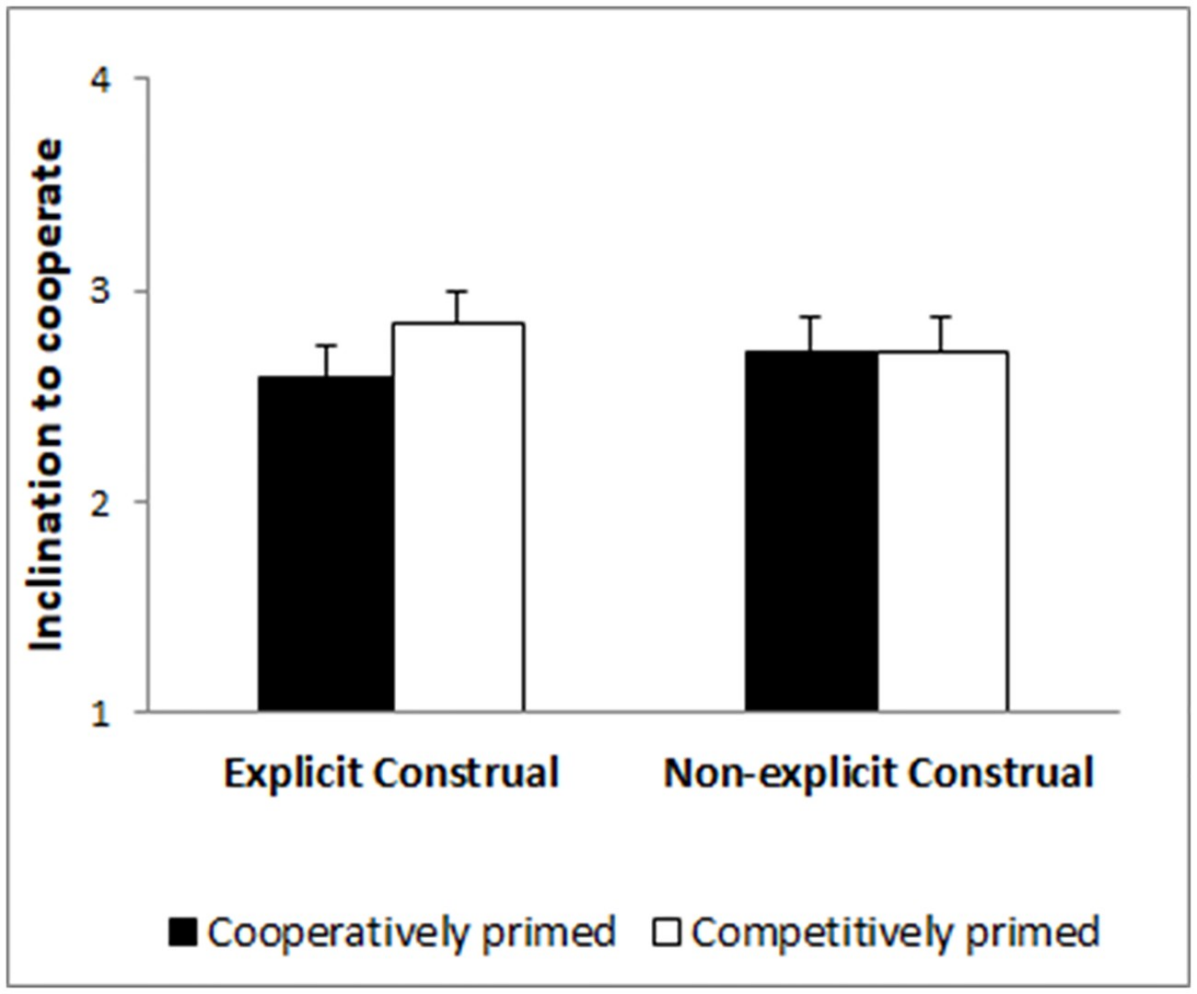
Intention to cooperate as a function of priming and construal order conditions.

Last, a 2x2 ANOVA of participants’ own behavioral intentions in the PD showed a non-significant effect of priming (*F*(1, 340) = 0.401, *p* = .527), a non-significant effect of construal order (*F*(1, 340) = 0.022, *p* = .883), and a non-significant priming x construal order interaction (*F*(1, 340) = 0.310, *p* = .578; see Fig 2).

Second, we report the results after applying the exclusion criteria mentioned before. Based on the debriefing questionnaire, we excluded 6 participants (1.73%) who showed indications of awareness of the goal of the study or the relationship between tasks. In addition, 10 participants (2.89%) were excluded because they did not answer the questions that allowed us to estimate their awareness. Furthermore, based on the control comprehension questions of the PD, 139 subjects (40%) were excluded because they had at least one incorrect answer. Importantly, results remained qualitatively the same regardless of whether analyses were done with or without excluding participants, as described next.

The sample after excluding participants (hereafter, “reduced sample”) consisted of 200 participants (age in years *M* = 21.36, *SD* = 3; 62.5% women).

In the reduced sample, we found again significant correlations of participants’ ratings of cooperative names (*r* = .359, *p* = .000), as well as of ratings of competitive names (*r* = .449, *p* = .000). The correlation of the composite measure of name appropriateness and beliefs about others’ intentions was also positive (*r* = .156, *p* = .029). However, 2x2 ANOVAs for the name rating composite measure and beliefs about others’ intention showed all no significant terms (all *p*s > .05).

Last, the 2x2 ANOVA of participants’ own behavioral intentions in the PD with the reduced sample showed again non-significant effects of priming (*F*(1, 196) = 0.306, *p* = .581), construal order (*F*(1, 196) = 0.103, *p* = .748), and priming x construal order interaction (*F*(1, 196) = 0.383, *p* = .536).

Third, we checked whether gender could have modulated present results. Some authors argue that gender may affect decisions in economic games. More specifically, it has been proposed that women are more sensitive to contextual cues than men [53]. According to this, the interaction between priming and construal order, or any of their main effects could work differently for women than for men. For instance, women could be more responsive to priming stimuli. This is relevant given that the percentage of women in the sample from Kay and Ross’ study (45%) was different from the percentage in the present sample (61%; Fisher’s exact test, *p* < .05), and, hence, differences between the gender compositions of the samples could underlie differences in the results between the original study and the present replication. Unfortunately, the data set of the original study is not available and, therefore, we cannot check for a potential gender effect in the original results. Nevertheless, we did assess the effect of gender in the present data by running 2x2x2 ANOVAs on the three main dependent variables (name appropriateness rating, beliefs about others’ intentions in the PD, and participants’ own behavioral intention in the PD) with priming, construal, and gender as fixed factors. Results showed no significant effect of gender as assessed in its main effects or in its interactions on any of the three dependent variables evaluated (all *p*s > .05).

Finally, in the appendix, we present the description of an independent experiment (*N* = 110), in which we tested the effect of the cooperative/competitive priming only in the condition with the construal task before the decision. In contrast with the experiment described above and Kay and Ross’s original procedure, we monetarily incentivized the PD decision in the experiment described in the appendix, but, again, we found no priming effect on the name rating task, participant’s beliefs about other’s decisions, or participants’ own decisions (see the S1 File for further details).

## Discussion

In a much cited paper from 2003, Kay and Ross [11] reported that US undergraduates showed more or less cooperative intentions in a Prisoner’s Dilemma (PD) after being primed with cooperative or competitive words, respectively. In addition, they reported a priming effect on participants’ construal of the situation as evidenced by their ratings of appropriateness for cooperative and competitive names for the game, and participants’ beliefs about others’ intention in the PD. Cooperative (relative to competitive) priming led to higher ratings of appropriateness for cooperative than competitive names, and also to beliefs that others would show a higher intention to cooperate. Last but not least, the priming effect on participants’ own behavioral intention was indeed stronger when their decision in the PD was completed after than before the name rating and beliefs elicitation tasks, suggesting that the explicit construal of the situation affected (intended) decisions in that context.

As mentioned in the introduction, Kay and Ross’ results may appear unsurprising and even intuitive, but they were found using a rather weak experimental design, due to a very small sample size. Whereas most attention in the psychological literature has been directed at replicating novel and surprising results, we believe it is of great importance to also evaluate findings that may have been easily accepted by the scientific community because they match common-sense or intuitive views, even when there is not enough or reliable evidence in their support.

In the study reported here, we ran a close replication of Kay and Ross’ 2x2 experimental design, with a sample size which secured sufficient power to replicate the original effects reported by these authors [11]. However, present results did not provide evidence in support of Kay and Ross´ original findings. Furthermore, we could not find evidence of any effect of priming cooperation/competition on monetarily incentivized decisions in the PD as detailed in the Appendix. We are thus left with the challenge of explaining why we did not replicate Kay and Ross’ findings. Though we recognize we cannot give a definitive answer in this respect, we explore some possible explanations next.

First, it is relevant to focus on a major limitation of the present replication attempt, namely that, beyond the descriptions in the paper, we did not count with the original materials or detailed descriptions of the procedures. The authors of the original study were willing to help but, unfortunately, did not have the materials and data stored, and were unable to recall the details. Therefore, we had to fill the gaps in the information obtained from the original paper with our best informed guesses. Consequently, there could be unknown methodological discrepancies between Kay and Ross´ experiment and the present study, which could explain the divergent results. For instance, it has been suggested that the presence of the researcher in the experimental room might make participants focus on this person instead of on the intended target of the prime, which could affect priming effects [33]. Due to a lack of details about the original study, it is no possible to examine whether a difference of this sort could explain differences in our results. This situation highlights the importance of registering and sharing the materials along with paper submissions [34].

Second, a factor to consider is the possible influence of time and cultural discrepancies between studies. The original study was published 13 years before the present replication was done, and in a different country (USA) with a different language (English). Nonetheless, to make sure that we were testing the alleged effect, we took great care to rely on priming stimuli that were culturally valid for the Argentine sample used (see preliminary studies). This agrees with Sroebe and Strack [47] who argue that simply duplicating a study’s procedure is not guarantee of addressing the same theoretical construct as the original study. In some cases, materials need to be modified to faithfully represent the intended constructs. This is especially true for a study that relies on subtly activating specific semantic categories (cooperation/competition). Consequently, following cautions on priming sensitivity to the testing context (e.g., [34, 30, 40, 54]), we took special care on pre-evaluating the priming stimuli and tasks, as it has been strongly recommended in the literature [47, 55]. We conducted two preliminary studies to make sure that the words used in the priming and name rating tasks were appropriate in representing the target concepts (cooperation and competition) for the population from which we drew our sample (undergrad students from the National University of Cordoba). Despite these precautions, we concede that a potential cultural effect of how people associate competition or cooperation with their own economic wellbeing could be the cause of differences between studies.

Third, another potential unmeasured influence on results could relate to discrepancies between studies in the distribution of participants´ individual differences, such as dispositional factors or gender proportions. For instance, the Social Value Orientation (SVO) has been reported as mediating the effect of competitive primes [56], and morality or might primes [57] in social dilemmas. Therefore, differences in SVO distributions between the original study and the present experiment could underlie differences in results. Nonetheless, a recent study showed similar distributions of SVOs in university students from Argentina and the USA (where the original study was done) [58], thus suggesting that strong divergences in SVOs between studies seems an unlikely possibility. Another difference between the original study and the present replication is gender proportions in the samples used. Whereas Kay and Ross [11] had a sample with a majority of men, the present sample had a majority of women. Although the evidence about gender effects on decision in economic games is not conclusive, a large review on gender preferences suggests that women are more risk averse, tend to avoid competitive situations, and cooperate at a higher rate than men [53]. Based on those findings, one may wonder whether gender composition differences between studies could explain different sensitivities to the cooperative or the competitive priming. However, in the same review, authors presented results revealing that women’s economic choices are more sensitive to social cues than men’s decisions. In fact, according to them, most gender differences on preferences can be explained by women’s greater sensitivity to the social context. Consequently, it could be expected that the priming effect should be stronger in a sample with a majority of women. This however contrasts with the comparison of results between Kay and Ross’ study and the presently described experiment. Whereas the original study had a slight majority of men and found a priming effect, the experiment described here had a majority of women but provided no evidence for the original effect. Furthermore, we could not find any significant main effect of gender or its interactions with priming and situational construal order on name ratings, beliefs about others’ intentions, or participants’ own behavioral intentions in the PD in the present sample. This evidence suggests gender to be mostly irrelevant in the present context, and, therefore, gender differences do not seem to be a likely candidate to account for different results between the original and present studies.

Finally, we need to consider the possibility that the original findings were a false positive. In this respect, we analyze the relevance of different factors. For instance, Kay and Ross [11] did not detail the procedure for selecting and designing their priming stimuli. Depending on the rigor of the procedure used, there is a possibility that the employed primes could have been interpreted or related to different concepts than those intended by the authors [52]. Additionally, from the methods reported in the original paper, it seems possible that the comprehension of the PD was not evaluated in the original study. If participants did not understand the game properly, chances are that their answers do not really relate to their intentions and preferences. In fact, reports in the experimental economics literature have shown different dispositions to cooperate in participants with different levels of understanding of the game [16]. Although the main results remained qualitatively unmodified when including or excluding subjects with insufficient comprehension in the present experiment, checking the understanding of game rules and payoffs is a necessary caution to minimize the influence of confusion. Last but not least, the sample size of the original study was very small, leading to a high risk of a Type 1 error [59–60]. Further analyses in the original data set could have shed some light on the accurateness of the original findings, and their comparability with present results. However, Kay and Ross do not have a record of their data and the details of results in their original paper are rather insufficient, lacking reports on standard deviations and confidence intervals. In any case, if real effect sizes were of a magnitude similar to the effects reported in the original study, the present replication would have a 99% probability of finding similar results with the complete sample (94% with the reduced sample). Despite being a very high probability, this number also means that there was a 1% (6% with the reduced sample) chance that we just missed the effect.

Before the end of this discussion, we believe it is important to clarify why we believe present experiments (i.e., including the experiment in the appendix) can be considered “close” or “direct” replications, and not “conceptual” replications, of Kay and Ross’ original experiment. There are diverse suggestions for classifying replication studies based on different criteria. Among them, Schmidt’s [32] classification is one of the most cited. According to this author, direct replications include studies that maintain the primary information (i.e., how the independent variable is presented to participants) as similar as possible to the original study, while elements from the contextual background and/or the procedure for the constitution of the dependent variable may vary. In contrast to direct replications, Schmidt [32] proposes that, in a conceptual replication, “one needs to construct a different experimental setup that conveys the same primary information focus by a radically different material realization” (p. 94). Similarly, Simons [61] argues that direct replications from different laboratories are crucial to separate the signal of interest from the noise, conceiving differences in the samples among studies as systematic noise, and measurement error as unsystematic noise. Makel, Plucker, and Hegarty [62], in turn, state that direct replications will inevitable change some aspects from the original study due to uncontrollable features, such as, for example, the passage of time. Therefore, the reduction of background changes to a minimum is what characterizes direct replications; though this does not necessarily mean that direct replications portray an *exact* reproduction of materials. In sum, we believe that “direct replication” best characterizes the present reproduction attempt because we recreated the primary information from the original study as close as possible.

The main explicit difference between Kay and Ross’ original study [11] and ours relates to the country of origin of the samples used (USA and Argentina, respectively). As mentioned before, Simons proposes that differences in characteristics of the samples can be considered as systematic noise, beyond the effect signal of interest, which, in the present case, was to test whether a competitive/cooperative priming influenced the perception of and behavioral intentions in a PD. Calling the present experiment a “direct replication” does not imply assuming that US and Argentine participants perceived and construed the reality of the game in the same way. What we argue here, however, is that the effect proposed in the original study has been examined by closely reproducing what was stated (both theoretically and methodologically) as relevant in the original paper. As Simons [61] put it: “The idea that direct replication undergirds science has a simple premise: If an effect is real and robust, any competent researcher should be able to obtain it when using the same procedures with adequate statistical power” (p. 76). Furthermore, other widely known direct replication projects also relied on samples which country of origin differed from that of the sample in the original study. This can be seen in the seminal work of the Open Science Collaboration [31], in which of the 100 psychological experiments replicated, there were some run with samples from a different country and a different language than the original study. Camerer et al.’s replication [63] of 18 experiments in experimental economics also present such cases. Therefore, many researchers seem to believe that changing the sampling country does not invalidate referring to a replication as “direct”.

In any case, as many authors have suggested (e.g., [64, 65]), replications constitute a spectrum rather than clear-cut set of categories. A noticeable recommendation has been to explicitly identify similarities and differences (e.g., context, cultural background, sample, setting) between replications and the original study in order to recognize where the replication stands in the continuum from ‘close’ to ‘conceptual’ [65]. From that perspective, we consider that both replications in the present article can be placed on the ‘close’ side of the spectrum, although the experiment in the main test is ‘closer’ to the original study than the replication described in the appendix.

To finish, we want to adequately frame the implications of present findings. Because of the impossibility of conducting an exact reproduction of a study, no replication is conclusive on its own. It is the aggregation of replication attempts what allows evaluating an effect and estimating effect sizes with further precision. Here we conducted two replications and found no evidence of the original findings. Indeed, the combination of results from both replications increases the bases for believing that the original findings, despite intuitive, could be a false positive. However, further replications, both direct and conceptual, would be needed to get a deeper understanding of this prime-to-behavior effect [66], and which contextual circumstances, unattended here, may explain its (dis)appearance [33, 44].

## Supporting information

S1 File Appendix. A complementary replication with monetarily incentivized decisions.
(DOCX)

S2 File Preliminary studies. Description of preliminary studies.
(DOCX)
