# Supplementary material for: Testing the effect of cooperative/competitive priming on the Prisoner’s Dilemma. A replication study
Source: PLoS One. 2018 Dec 20;13(12):e0209263. doi: 10.1371/journal.pone.0209263 (PMC6301569; doi:10.1371/journal.pone.0209263)
Supplement: S1 File — A complementary replication with monetarily incentivized decisions. (DOCX) [file pone.0209263.s001.docx]

S1 Appendix: A complementary replication with monetarily incentivized decisions

Anabel Belaus, Cecilia Reyna, Esteban Freidin.

**The experiment**

**Materials and methods**

**Participants**

For replications, Simonsohn [1] suggests a sample size at least 2.5 times larger than the original to have 80% power to reject an effect size of 0.5, if the true effect is zero. Kay and Ross [2] included 56 participants in their 2x2 design. However, in this experiment, we attempted to replicate only 2 of the 4 treatments done by them; hence, we considered *n*=28 (14 participants per treatment) in the original study, and we aimed for a sample size of at least 70 participants for the present experiment.

A total of 110 students from the National University of Cordoba, Argentina, participated in this study (age range 18-35years old; mean 22.6; 63.6%women). We ran 10 sessions, each having between 9 to 14 participants. Subjects participated only once and had not taken part in the preliminary studies detailed in the main text.

All participants signed a written consent form for participating in the study, and present protocols were reviewed and approved by the Bioethical Committee of the Hospital Municipal “Dr. Leónidas Lucero”, Bahía Blanca, Argentina.

**Materials and design**

The priming manipulation was performed using the Scrambled-sentence task described in the main text. This task consisted of 24 non-grammatical five-word sentences that participants had to rearrange into grammatically coherent four-word sentences. Out of the 24 sentences, 16 included words related to the priming treatment, and the remaining sentences were intended to be neutral in terms of their relationship with the concepts of cooperation and competition. Half of the participants received the cooperative primed sentences, while the other half completed competitive primed sentences. To adapt the scrambled sentences to the Argentinean population, we conducted three preliminary studies (see the *Methods* section in the main text for the details).

The tasks for the situational construal evaluation and decision in the PD game were translated to Spanish from the text used in the original article. However, given that we did not count with the complete set of original materials, the general instructions were created from our best judgment. The situational construal tasks were identical to the ones described in the main text. In contrast, the decision task was different. First, in the experiment described in the main text, decision payoffs were hypothetical, whereas in the present experiment, participants´ decisions could have monetary consequences for them and a randomly determined and anonymous associated participant in the session. Second, whereas in the experiment described in the main text, participants were asked for their behavioral intention in the PD (scaled from 1:“I will surely choose A” to 5: “I will surely choose B”), in this experiment, participants had to choose between cooperating (choose A) or defecting (choose B) with their associated participant.

**Procedure**

The procedure followed was exactly the same as that described in the main text, with the following exceptions: 1) After participants read and signed the consent form, each received a raffle number to individually identify them without tracking their real identities; this number was used to determine payoffs and delivering the cash payment at the end of the session; and 2) at the end of each session (as they had been told in the oral instructions in the beginning of the session), two randomly selected participants received a monetary payment according to the combination of their decisions in the PD. Participants also knew that to be selectable for payment, all comprehension questions had to be answered correctly; otherwise, a new random participant would be selected. Once two participants with correct responses in all comprehension questions were selected, their PD decisions were combined and their payment calculated. This task was performed by a collaborator outside the experimental room to make sure the experimenters could not associate participants’ faces with their earnings. Also to warrant the anonymity of those selected for payment, all participants received an envelope with a sheet of paper inside so that envelopes with and without cash could not be told apart from the outside. Each participant collected the envelope with their corresponding raffle number from a table near the exit door on their way out.

**Results**

First, we describe the results from analyses with the full sample (*N*=110). Second, we report results after applying the exclusion criteria mentioned in the main text (*n*=69).

In correspondence with Kay and Ross procedure, we first examined the correlation between participants’ rating of cooperative names (*r* = .335, *p <* .001) and the correlation between ratings of competitive names (*r* = .405, *p <* .001). Given these significant correlations, we combined the ratings of two cooperative names and those of the two competitive names, obtaining an overall cooperative name rating and an overall competitive name rating, respectively. With these scores, we generated a composite measure of “relative appropriateness” by subtracting the overall competitive measure from the overall cooperative measure. A higher score in the composite measure indicated a “more cooperative construal of the situation” [2, pp. 683]. Contrary to Kay and Ross´ results, we did not find evidence of a significant correlation between the composite measure and beliefs about others´ cooperative/competitive decisions in the PD (*r* = -.054, *p* = .578).

Whereas the effect of priming on the composite measure of name rating was non-significant (*F*(1, 107) = 0.164, *p* = .686), the effect of priming on beliefs was statistically significant (*F*(1, 108) = 5.559, *p* = .020; Cooperation: *M* = 49.38, *SD* = 27.58; Competition: *M* = 60.58, *SD* = 21.91, Cohen’s *d* = -.45). Regarding the priming effect on decisions, we found that about less than half of the participants cooperated in the cooperative condition (25 out of 55, 46%), whereas the majority decided to cooperate in the competitive condition (36 out of 55, 65%). Though this pattern is opposite to that found by Kay and Ross, the difference in decisions between conditions was only marginally significant (Fisher´s exact test, *p* = .055).

In the debriefing questionnaire, 4 participants expressed awareness of the goal of the study or the relationship between tasks, and 1 participant did not answer enough questions to allow the evaluation of the awareness level. In turn, 36 participants answered incorrectly at least one of the comprehension questions. Consequently, a total of 41 participants were discarded from the sample (hereafter, “reduced sample”), thus leaving 69 subjects (35 in the Cooperative treatment, and 34 in the Competitive treatment).

In the reduced sample, the correlation between participants’ rating of cooperative names (*r* = .273, *p* = .023) and the correlation between ratings of competitive names (*r* = .499, *p* = .000) were again both significant, thus allowing to build the composite name rating composite measure as explained before. Again, we could not find evidence of a significant correlation between the composite measure and beliefs about others´ cooperative/competitive decisions in the PD (*r* = -.034, *p* = .783).

When we examined the treatment effect on the name rating task in the reduced sample, results showed no significant effect of priming (*F*(3, 64) = 1.62, *p* = .581, *η²_p_*= .075). We found no evidence of a priming effect on cooperative names, in competitive names, or in the neutral name (see Table 1 on this appendix). Despite significant effects of priming on beliefs and decisions when the full sample was considered, we did not find evidence of a priming effect on beliefs or decisions in the reduced sample (see Table 1). Nonetheless, it is worth noting that average differences between treatments for both beliefs and decisions were in the same direction than when the full sample was considered, and therefore, in the opposite direction than Kay and Ross´ results. Importantly, no statistical differences were found between participants who answered all the comprehension questions correctly and those who did not, neither for beliefs about other participants’ decisions (*t*(103) = 1.66, *p* = .099, Cohen’s *d* = .033), nor for their own decisions (𝟀^2^(1) = .002, *p* = .962, *ɸ* = .004).

Table 1. Priming effects on name rating, beliefs about other participants’ behavior, and decision on the Prisoner’s Dilemma game.

|  | **Cooperation condition** | | | |  | **Competition condition** | | | |  | **Conditions comparison** | | | |
| --- | --- | --- | --- | --- | --- | --- | --- | --- | --- | --- | --- | --- | --- | --- |
|  | n | *M* | *SD* | 95% *CI* |  | n | *M* | *SD* | 95% *CI* |  | *Statistic*(df)=value | 95% *CI* | *p* | Effect size |
| Rating of Cooperative names | 35 | 11.03 | 4.62 | 9.47 – 12.58 |  | 33 | 9.61 | 4.58 | 8 – 11.2 |  | *F*(1, 66) = 1.62 | -3.65, .808 | .207 | *η²_p_* = .023 |
| Rating of Competitive names | 35 | 6.91 | 4.42 | 5.45 – 8.4 |  | 33 | 7.85 | 4.40 | 6.31 – 9.38 |  | *F*(1, 66) = .763 | -1.20, 3.07 | .386 | *η²_p_* = .011 |
| Rating of Neutral name | 35 | 6.46 | 2.64 | 5.55 – 7.36 |  | 33 | 6.18 | 2.72 | 5.25 – 7.11 |  | *F*(1, 66) = .179 | -1.57, 1.02 | .673 | *η²_p_* = .002 |
| Others´ expected cooperation | 35 | 49.14 | 26.88 | 39.91 – 58.47 |  | 34 | 55.38 | 22.36 | 47.58 – 63.19 |  | *t*(67) = -1.05 | -5.65, 18.13 | .299 | *d* = .25 |
| Cooperative choice | 35 | 16 (45.71%) | | |  | 34 | 22 (64.7%) | | |  | 𝟀*^2^*(1) = 2.51 | ---- | .113 | *ɸ* = .191 |

**Discussion**

In this replication, we found no reliable evidence of an effect of priming cooperation/competition on the perception of the PD, the beliefs about others’ behavior in the game, or participants´ own cooperative decisions. The results showed *p*-values above the standard alpha level (α = .05) and small effect sizes. When including the whole sample (i.e., without discarding participants for lack of comprehension or awareness of the priming intention), some statistical indicators changed, but not in a qualitative way. These changes may be interpreted as due to the increase in the sample size and/or as consequence of considering responses from participants that seemingly lacked a proper understanding of the game. However, considering the absence of significant differences between participants who answered all the comprehension questions correctly and those who did not, the difference between indicators with and without including the whole sample is more likely due to the change in the sample size. In any case, these indicators should be interpreted with caution.

Although results did not indicate significant differences between priming conditions, it is striking to observe a tendency in the opposite direction than expected based on Kay and Ross´ results [2]. While we anticipated a priming assimilation effect, present data agree more with a contrast effect. Contrast effects have been suggested to originate from comparison processes [8, 10], such as comparing the self with a prototype (e.g., [10-11]). However, the present design closely resembles that of Kay and Ross [2], and there is no evident characteristic on their design which may lead to expect a comparison process. In fact, their design and, consequently ours, had the necessary elements for an assimilation effect [12]: 1) the PD game was described ambiguously and framed neutrally, that is, without explicit content “of a specific social situation” ([12], pp. 279); and 2)participants’ attention was explicitly guided towards the game, as they completed the PD tasks immediately after the priming task, and there were no other distracting activities to which the primes might have been perceived as relevant or applying to. The main difference between the original and the present procedure was the implementation of economic incentives for the decision in the present PD. However, to our best knowledge, there is no evidence of monetary payments inducing contrast effects. In fact, several priming studies found assimilation effects while employing economically incentivized decisions (e.g., [13 - 15]).

Finally, it is important to note that although we increased the sample size 2.46 times relative to the original study, we did not achieve enough statistical power in the present experiment which was 17% for beliefs about others’ behavior, and 35% for decisions (post-hoc calculations done in G*Power [16]).

**References**

1. Simonsohn U. Small Telescopes. Psychological Science. 2015; 26(5):559–69.

2. Kay AC, Ross L. The perceptual push: The interplay of implicit cues and explicit situational construals on behavioral intentions in the Prisoner’s Dilemma. Journal of Experimental Social Psychology. 2003; 39(6):634–43.

3. Srull TK, Wyer RS. The role of category accessibility in the interpretation of information about persons: Some determinants and implications. Journal of Personality and Social Psychology. 1979; 37(10): 1660–72.

4. Hertwig R, Ortmann A. Experimental practices in economics: A challenge for psychologists? Behavioral And Brain Sciences. 2001; 24, 383–451.

5. Zizzo DJ. Experimenter Demand Effects in Economic Experiments. Experimental Economics.2010; 13:75–98

6. Feltovich N. What’s to know about laboratory Experimentation in economics? Journal of Economic Surveys. 2011; 25(2): 371–379.

7. Rand DG, Newman GE, Wurzbacher OM. Social Context and the Dynamics of Cooperative Choice. Journal of Behavioral Decision Making. 2014Dec;28(2):159–66

8. Bargh JA, Chartrands TL. The mind in the middle. A practical guide to priming and automaticity research. In: Reis H, Judd C editors. Handbook of research methods in social psychology. New York: Cambridge University Press. 2000. Pp. 253-285.

9. Rand DG. Cooperation, Fast and Slow.Psychological Science. 2016; 27(9): 1192–206.

10. Dijksterhuis A, Spears R, Lépinasse V. Reflecting and Deflecting Stereotypes: Assimilation and Contrast in Impression Formation and Automatic Behavior. Journal of Experimental Social Psychology. 2001;37(4):286–99.

11. Aarts H, Dijksterhuis A. Category activation effects in judgment and behavior: The moderating role of perceived comparability. British Journal of Social Psychology.2002; 41:123–138.

12. Kay AC, Wheeler SC, Smeesters D. The situated person: Effects of construct accessibility on situation construals and interpersonal perception. Journal of Experimental Social Psychology. 2008;44(2):275–91.

13. Shariff AF, Norenzayan A. God Is Watching You. Priming God Concepts Increases Prosocial Behavior in an Anonymous Economic Game Psychological Science. 2007;18(9):803–9

14. Capraro V, Smyth C, Mylona K, Niblo GA. Benevolent Characteristics Promote Cooperative Behaviour among Humans. PLoS ONE.2014; 9(8): e102881.

15. Gu R, Yang J, Shi Y, Luo Y, Luo YLL, Cai H. Be Strong Enough to Say No: Self-Affirmation Increases Rejection to Unfair Offers. Frontiers in Psychology.2016; 7: 1824.

16. G*Power, 2009Buchner A, Erdfelder E, Faul F, Lang A. G*Power (Version 3.1.2) [Computer program]. 2009 Available from: http://www.psycho.uni-duesseldorf.de/aap/projects/gpower/.

**Supporting information**

Complete project at: <https://osf.io/dhfns/>- doi: 10.17605/OSF.IO/DHFNS

Supplementary materialS3 **Materials, data, and scripts for First Replication**osf.io/7rncj
