# Supplementary material for: Testing the effect of cooperative/competitive priming on the Prisoner’s Dilemma. A replication study
Source: PLoS One. 2018 Dec 20;13(12):e0209263. doi: 10.1371/journal.pone.0209263 (PMC6301569; doi:10.1371/journal.pone.0209263)
Supplement: S2 File — Description of preliminary studies. (DOCX) [file pone.0209263.s002.docx]

Testing the effect of cooperative/competitive priming on the Prisoner’s Dilemma. A replication study.

Belaus, Anabel, Reyna, Cecilia, & Freidin, Esteban.

Preliminary studies

Kay and Ross (2003) evaluated the effect of cooperative/competitive primes and the mediational influence of a situational construal task (i.e., asking participants to assess the situation as cooperative or competitive) on the Prisoner's Dilemma (PD) game. Their core hypothesis was that presenting words about cooperation/competition would make a person's thoughts about cooperativeness/competitiveness more cognitively available. Then, when judging an ambiguous situation like the PD, that person would more likely perceive the situation as cooperative/competitive. The same would happen when asked to state her beliefs about others´ expected behavior in the situation, and when making her own decision in that context. Indeed, Kay and Ross findings supported these hypotheses: participants in the cooperative condition considered cooperative names as more appropriate for naming the PD game, expected more cooperation from others, and expressed a higher intention to cooperate themselves than participants in the competitive condition.

We aimed at replicating Kay and Ross´s (2003) study on the effect of priming cooperation/competition over the perception of and decisions in a PD game. In this document, we detailed three preliminary studies conducted to adapt to our culture and language the scrambled-sentence task employed in the original research for the priming manipulation.

**Preliminary study 1**

The goal of first preliminary study was to create a list of words perceived as related to cooperation or competition in the local population of university students. It was conducted online through a LimeSurvey platform with the National University of Cordoba license. Participants were invited through email and announcements in social media related to university life.The task consisted of writing down as many words as possible related to the target word. There were two between-subject conditions: in the Cooperation condition the word cooperation was shown as a target (n = 52), whereas in the Competition condition the word competition was shown as a target (n = 46).There was no time limit to complete the task. Finally, some basic socio-demographic data was requested.

A total of 98 students from the National University of Cordoba with age range 18 to 35 years old completed the study, which was available online from February 28th to March 1st 2017.

To analyze the answers, we transformed each word into infinitive, singular, and masculine. We also checked for spelling errors and eliminated answers which did not respond to the task. All the analyses were performed using the resulting words. We obtained a total of 169 words as related to cooperation (see Table 1), and 194 related to competition (see Table 2). We calculated the frequency of each word and generated a rank for each condition. A detailed list of words, the frequency of appearance, and ranking are presented in Table 1 and Table 2 for cooperative and competitive conditions respectively. We also calculated the frequency with which present participants mentioned the words used in the original research done Kay and Ross (2003) and some synonyms of them (see Table 3 for the cooperative condition; see Table 4 for the competitive condition). A complete analytic dataset is available on osf.io/m9gh2.

**Table 1**. List, frequency, and ranking of words related to cooperation.

| **Word** | **Frequency** | **Ranking** |
| --- | --- | --- |
| ayuda | 39 | 1 |
| solidaridad | 24 | 2 |
| equipo | 21 | 3 |
| trabajo | 15 | 4 |
| compañerismo | 13 | 5 |
| grupo | 13 | 5 |
| amistad | 12 | 7 |
| colaborar | 10 | 8 |
| comunidad | 7 | 9 |
| conjunto | 7 | 9 |
| union | 7 | 9 |
| objetivo | 6 | 12 |
| empatia | 5 | 13 |
| participar | 5 | 13 |
| sociedad | 5 | 13 |
| cooperativa | 4 | 16 |
| organizacion | 4 | 16 |
| acuerdo | 3 | 18 |
| aportar | 3 | 18 |
| comprension | 3 | 18 |
| compromiso | 3 | 18 |
| juntos | 3 | 18 |
| mutuo | 3 | 18 |
| persona | 3 | 18 |
| progreso | 3 | 18 |
| relacion | 3 | 18 |
| accion | 2 | 27 |
| amor | 2 | 27 |
| apoyo | 2 | 27 |
| asistir | 2 | 27 |
| asociacion | 2 | 27 |
| bienestar | 2 | 27 |
| capitalismo | 2 | 27 |
| colaboracion | 2 | 27 |
| colectivo | 2 | 27 |
| comun | 2 | 27 |
| contribuir | 2 | 27 |
| cooperacion | 2 | 27 |
| coordinacion | 2 | 27 |
| crecimiento | 2 | 27 |
| esfuerzo | 2 | 27 |
| facilitar | 2 | 27 |
| familia | 2 | 27 |
| horizontalidad | 2 | 27 |
| humano | 2 | 27 |
| integrar | 2 | 27 |
| necesidad | 2 | 27 |
| orden | 2 | 27 |
| reciprocidad | 2 | 27 |
| respeto | 2 | 27 |
| responsabilidad | 2 | 27 |
| social | 2 | 27 |
| socialismo | 2 | 27 |
| voluntad | 2 | 27 |
| aceptar | 1 | 55 |
| actividad | 1 | 55 |
| agradecer | 1 | 55 |
| agrupacion | 1 | 55 |
| altruismo | 1 | 55 |
| amabilidad | 1 | 55 |
| ambiente | 1 | 55 |
| atencion | 1 | 55 |
| autogestion | 1 | 55 |
| ayuda mutua | 1 | 55 |
| bien comun | 1 | 55 |
| bilateral | 1 | 55 |
| brindar | 1 | 55 |
| buena onda | 1 | 55 |
| camaraderia | 1 | 55 |
| caridad | 1 | 55 |
| cariño | 1 | 55 |
| carrera | 1 | 55 |
| causa | 1 | 55 |
| club | 1 | 55 |
| compañia | 1 | 55 |
| comunion | 1 | 55 |
| conciencia | 1 | 55 |
| confianza | 1 | 55 |
| construccion | 1 | 55 |
| conveniencia | 1 | 55 |
| convivencia | 1 | 55 |
| cooperadora | 1 | 55 |
| critica | 1 | 55 |
| deber | 1 | 55 |
| dedicacion | 1 | 55 |
| desarrollo | 1 | 55 |
| desinteres | 1 | 55 |
| desventaja | 1 | 55 |
| diferencia | 1 | 55 |
| disponibilidad | 1 | 55 |
| distribucion | 1 | 55 |
| dos | 1 | 55 |
| educacion | 1 | 55 |
| eficiencia | 1 | 55 |
| encuentro | 1 | 55 |
| entusiasmo | 1 | 55 |
| estado | 1 | 55 |
| estar presente | 1 | 55 |
| estrategia | 1 | 55 |
| evolucion | 1 | 55 |
| fin | 1 | 55 |
| fraternidad | 1 | 55 |
| ganador | 1 | 55 |
| generosidad | 1 | 55 |
| gente | 1 | 55 |
| gentil | 1 | 55 |
| hacer | 1 | 55 |
| humanidad | 1 | 55 |
| humildad | 1 | 55 |
| ideas | 1 | 55 |
| iglesia | 1 | 55 |
| igualdad | 1 | 55 |
| incluir | 1 | 55 |
| individualismo | 1 | 55 |
| individuo | 1 | 55 |
| instituto | 1 | 55 |
| integridad | 1 | 55 |
| interaccion | 1 | 55 |
| intercambio | 1 | 55 |
| interes | 1 | 55 |
| internacional | 1 | 55 |
| intervencion | 1 | 55 |
| lazo | 1 | 55 |
| lealtad | 1 | 55 |
| lider | 1 | 55 |
| logro | 1 | 55 |
| lucha | 1 | 55 |
| mancomunar | 1 | 55 |
| manos | 1 | 55 |
| mantenimiento | 1 | 55 |
| meta | 1 | 55 |
| moral | 1 | 55 |
| muchos | 1 | 55 |
| mujeres | 1 | 55 |
| mundo | 1 | 55 |
| naturaleza | 1 | 55 |
| necesario | 1 | 55 |
| negociacion | 1 | 55 |
| neoliberalismo | 1 | 55 |
| noble | 1 | 55 |
| ong | 1 | 55 |
| oportunidad | 1 | 55 |
| par | 1 | 55 |
| paz | 1 | 55 |
| pensamiento | 1 | 55 |
| perdedor | 1 | 55 |
| preocupacion | 1 | 55 |
| problema | 1 | 55 |
| proceso | 1 | 55 |
| producir | 1 | 55 |
| progresar | 1 | 55 |
| projimo | 1 | 55 |
| pueblo | 1 | 55 |
| puntos de vista | 1 | 55 |
| racional | 1 | 55 |
| repartir | 1 | 55 |
| servicio | 1 | 55 |
| simpatia | 1 | 55 |
| sistema | 1 | 55 |
| solucion | 1 | 55 |
| sostener | 1 | 55 |
| sumar | 1 | 55 |
| trabajar juntos | 1 | 55 |
| transversalidad | 1 | 55 |
| unidad | 1 | 55 |
| valores | 1 | 55 |
| varones | 1 | 55 |
| voluntario | 1 | 55 |
| ayuda | 39 | 1 |

**Table 2**. List, frequency, and ranking of words related to competition.

| **Word** | **Frequency** | **Ranking** |
| --- | --- | --- |
| ganar | 19 | 1 |
| perder | 11 | 2 |
| esfuerzo | 9 | 3 |
| rivalidad | 9 | 3 |
| habilidad | 8 | 5 |
| equipo | 7 | 6 |
| capacidad | 6 | 7 |
| carrera | 6 | 7 |
| aptitud | 5 | 9 |
| conocimiento | 5 | 9 |
| deporte | 5 | 9 |
| meta | 5 | 9 |
| competencia | 4 | 13 |
| eficiencia | 4 | 13 |
| juego | 4 | 13 |
| lucha | 4 | 13 |
| premio | 4 | 13 |
| preparacion | 4 | 13 |
| triunfo | 4 | 13 |
| voluntad | 4 | 13 |
| derrota | 3 | 21 |
| desafio | 3 | 21 |
| egoismo | 3 | 21 |
| individualismo | 3 | 21 |
| participar | 3 | 21 |
| pelea | 3 | 21 |
| puesto | 3 | 21 |
| responsabilidad | 3 | 21 |
| superar | 3 | 21 |
| capitalismo | 2 | 30 |
| compañero | 2 | 30 |
| compromiso | 2 | 30 |
| confianza | 2 | 30 |
| dedicacion | 2 | 30 |
| desarrollo | 2 | 30 |
| enfrentamiento | 2 | 30 |
| estrategia | 2 | 30 |
| evento | 2 | 30 |
| fuerza | 2 | 30 |
| logro | 2 | 30 |
| maldad | 2 | 30 |
| medalla | 2 | 30 |
| muerte | 2 | 30 |
| orgullo | 2 | 30 |
| presion | 2 | 30 |
| rapido | 2 | 30 |
| sacrificio | 2 | 30 |
| superacion | 2 | 30 |
| tiempo | 2 | 30 |
| torneo | 2 | 30 |
| trampa | 2 | 30 |
| utilidad | 2 | 30 |
| velocidad | 2 | 30 |
| victoria | 2 | 30 |
| adecuado | 1 | 55 |
| animarse | 1 | 55 |
| ansiedad | 1 | 55 |
| aprehension | 1 | 55 |
| apremio | 1 | 55 |
| apropiado | 1 | 55 |
| atletismo | 1 | 55 |
| audacia | 1 | 55 |
| automovilismo | 1 | 55 |
| ayuda | 1 | 55 |
| bandera | 1 | 55 |
| batir | 1 | 55 |
| calificacion | 1 | 55 |
| campeon | 1 | 55 |
| capaz | 1 | 55 |
| carisma | 1 | 55 |
| civilizacion | 1 | 55 |
| codicia | 1 | 55 |
| codigo procesal | 1 | 55 |
| competente | 1 | 55 |
| complot | 1 | 55 |
| confrontacion | 1 | 55 |
| constancia | 1 | 55 |
| cooperacion | 1 | 55 |
| copa | 1 | 55 |
| correr | 1 | 55 |
| costumbre | 1 | 55 |
| cualidad | 1 | 55 |
| demostrar | 1 | 55 |
| desacuerdo | 1 | 55 |
| desempeño | 1 | 55 |
| desenvoltura | 1 | 55 |
| desestabilidad | 1 | 55 |
| desilucion | 1 | 55 |
| desinteres | 1 | 55 |
| desmotivacion | 1 | 55 |
| despejar mente | 1 | 55 |
| destacarse | 1 | 55 |
| desventaja | 1 | 55 |
| determinacion | 1 | 55 |
| dinero | 1 | 55 |
| disfrutar | 1 | 55 |
| dos | 1 | 55 |
| duelo | 1 | 55 |
| economia | 1 | 55 |
| eficacia | 1 | 55 |
| empeño | 1 | 55 |
| empresario | 1 | 55 |
| enemigo | 1 | 55 |
| entender | 1 | 55 |
| entrenar | 1 | 55 |
| entusiasmo | 1 | 55 |
| envidia | 1 | 55 |
| equidad | 1 | 55 |
| estudio | 1 | 55 |
| examen | 1 | 55 |
| exigencia | 1 | 55 |
| experiencia | 1 | 55 |
| facultad | 1 | 55 |
| falta de compañerismo | 1 | 55 |
| famaf | 1 | 55 |
| felicidad | 1 | 55 |
| formacion | 1 | 55 |
| frustracion | 1 | 55 |
| ganancia | 1 | 55 |
| ganas | 1 | 55 |
| grupo | 1 | 55 |
| guerra | 1 | 55 |
| hablar | 1 | 55 |
| heterogeneidad | 1 | 55 |
| humillacion | 1 | 55 |
| imposicion | 1 | 55 |
| incertidumbre | 1 | 55 |
| incompetencia | 1 | 55 |
| infringir | 1 | 55 |
| ingenio | 1 | 55 |
| instruccion | 1 | 55 |
| intensidad | 1 | 55 |
| intenso | 1 | 55 |
| juez | 1 | 55 |
| jugador | 1 | 55 |
| jurisdiccion | 1 | 55 |
| justicia | 1 | 55 |
| lider | 1 | 55 |
| logica | 1 | 55 |
| maximo | 1 | 55 |
| mejora | 1 | 55 |
| mejorar | 1 | 55 |
| merito | 1 | 55 |
| miame | 1 | 55 |
| miedo | 1 | 55 |
| modernidad | 1 | 55 |
| natacion | 1 | 55 |
| negocio | 1 | 55 |
| nota | 1 | 55 |
| objetivo | 1 | 55 |
| odio | 1 | 55 |
| olimpiada | 1 | 55 |
| olvido | 1 | 55 |
| oponente | 1 | 55 |
| optimismo | 1 | 55 |
| ovlidar | 1 | 55 |
| peligro | 1 | 55 |
| pensar | 1 | 55 |
| perseverancia | 1 | 55 |
| pesimismo | 1 | 55 |
| planear | 1 | 55 |
| plata | 1 | 55 |
| poder | 1 | 55 |
| podio | 1 | 55 |
| precision | 1 | 55 |
| profesional | 1 | 55 |
| recompensa | 1 | 55 |
| reir | 1 | 55 |
| resolucion | 1 | 55 |
| reto | 1 | 55 |
| riesgo | 1 | 55 |
| rigurosidad | 1 | 55 |
| riña | 1 | 55 |
| saber | 1 | 55 |
| saber hacer | 1 | 55 |
| sagaz | 1 | 55 |
| salvaje | 1 | 55 |
| seguir | 1 | 55 |
| seguridad | 1 | 55 |
| sentir | 1 | 55 |
| superioridad | 1 | 55 |
| supervivencia | 1 | 55 |
| talento | 1 | 55 |
| tarea | 1 | 55 |
| tecnica | 1 | 55 |
| tecnicidad | 1 | 55 |
| trabajo | 1 | 55 |
| tribunales | 1 | 55 |
| trivial | 1 | 55 |
| trofeo | 1 | 55 |
| valor | 1 | 55 |
| vencer | 1 | 55 |
| vergüenza | 1 | 55 |
| violencia | 1 | 55 |
|  |  |  |

**Table 3**.Cooperative words and synonyms from Kay and Ross´ (2003) study and their frequency of mentions in the present sample.

| **Word** | **Frequency** |
| --- | --- |
| ayuda | 39 |
| aliviar | 0 |
| armonía | 0 |
| paz | 1 |
| alianza | 0 |
| union | 7 |
| familia | 2 |
| justo | 0 |
| equitativo | 0 |
| imparcial | 0 |
| pacto | 0 |
| acuerdo | 3 |
| cariño | 1 |
| acojedor | 0 |
| calido | 0 |
| cooperacion | 0 |
| distribucion | 1 |
| repartir | 1 |
| razonable | 0 |
| sensato | 0 |
| decente | 0 |
| coincidir | 0 |
| acuerdo | 3 |
| aceptar | 1 |
| acceder | 0 |
| concordar | 0 |
| mutuo | 3 |
| reciproco | 0 |
| comun | 2 |
| compartir | 0 |
| hermandad | 0 |
| amistad | 12 |

**Table 4**.Competitive words and synonyms from Kay and Ross´ (2003) study and their frequency of mentions in the present sample.

| **Word** | **Frequency** |
| --- | --- |
| agresivo | 0 |
| beligerante | 0 |
| enemigo | 1 |
| ventaja | 0 |
| abogado | 0 |
| manipulacion | 0 |
| Darwin | 0 |
| competencia | 4 |
| poder | 1 |
| feroz | 0 |
| torneo | 2 |
| desconsiderado | 0 |
| perder | 11 |
| batalla | 0 |
| enfrentamiento | 2 |
| lucha | 4 |
| combate | 0 |
| sanguinario | 0 |
| violencia | 1 |
| capitalismo | 2 |
| desalmado | 0 |
| despiadado | 0 |
|  |  |

**Preliminary study 2**

The second preliminary study was conducted online with students from the National University of Cordoba (age range 18 to 35 years old), using the same platform as the previous preliminary study. The task consisted of scoring the closeness of a list of 30 words to the target word (cooperation or competition), using a 4 points scale (0 = “non-related at all” to 4 = “highly related”).The words included in the list were those from the original study (Kay & Ross, 2003) and the most frequent from the first preliminary study for the corresponding condition (cooperation, n = 51; competition, n = 75). The order of words’ presentation was randomized for each participant. A total of 126 students completed the task, which was available online from March 3rd to March 8th 2017. A complete analytic dataset is on osf.io/m9gh2.

We summed up the scores for each word to construct a ranking of closeness. Table 5 and 6 shows the total score for each word in the cooperation and the competition condition, respectively.

**Table 5**. Total score and ranking for words for the cooperative condition

| **Word** | **Total score** | **Ranking position** |
| --- | --- | --- |
| COOPERACIÓN* | 145 | 1 |
| EQUIPO | 133 | 2 |
| COLABORAR | 130 | 3 |
| PARTICIPAR | 117 | 4 |
| COMPAÑERISMO | 113 | 5 |
| AYUDA* | 112 | 6 |
| GRUPO | 111 | 7 |
| SOLIDARIDAD | 109 | 8 |
| UNIÓN* | 108 | 9 |
| COMUNIDAD | 105 | 10 |
| COMPARTIR* | 104 | 11 |
| ACUERDO* | 99 | 12 |
| MUTUO* | 95 | 13 |
| SOCIEDAD* | 95 | 13 |
| CONJUNTO | 91 | 15 |
| DISTRIBUCIÓN* | 90 | 16 |
| ARMONÍA* | 78 | 17 |
| EMPATÍA | 78 | 17 |
| HERMANDAD* | 75 | 19 |
| ACEPTAR* | 74 | 20 |
| COMÚN* | 71 | 21 |
| FAMILIA* | 69 | 22 |
| JUSTO* | 67 | 23 |
| REPARTIR* | 67 | 23 |
| PAZ* | 65 | 25 |
| RAZONABLE* | 64 | 26 |
| AMISTAD* | 63 | 27 |
| CARIÑO* | 42 | 28 |
| CÁLIDO* | 36 | 29 |
| IMPARCIAL* | 29 | 30 |

* words or synonyms from Kay and Ross´ (2003) study.

**Table 6**.Total score and ranking for words for the competitive condition.

| **Word** | **Total score** | **Ranking position** |
| --- | --- | --- |
| COMPETENCIA* | 209 | 1 |
| ESFUERZO | 186 | 2 |
| HABILIDAD | 185 | 3 |
| GANAR | 179 | 4 |
| DEPORTE | 171 | 5 |
| TORNEO | 170 | 6 |
| APTITUD | 169 | 7 |
| CAPACIDAD | 169 | 7 |
| RIVAL | 166 | 9 |
| CARRERA | 163 | 10 |
| ENFRENTAMIENTO* | 162 | 11 |
| META | 159 | 12 |
| EQUIPO | 154 | 13 |
| PERDER* | 154 | 13 |
| LUCHA* | 139 | 15 |
| CAPITALISMO* | 125 | 16 |
| COMBATE* | 121 | 17 |
| BATALLA* | 114 | 18 |
| VENTAJA* | 113 | 19 |
| PODER* | 112 | 20 |
| ENEMIGO* | 82 | 21 |
| AGRESIVO* | 78 | 22 |
| FEROZ* | 72 | 23 |
| DARWIN* | 69 | 24 |
| MANIPULACIÓN* | 67 | 25 |
| DESPIADADO* | 56 | 26 |
| VIOLENCIA* | 56 | 26 |
| ABOGADO* | 52 | 28 |
| SANGUINARIO* | 47 | 29 |
| DESCONSIDERADO* | 36 | 30 |

* wordsor synonyms from Kay and Ross´ (2003) study.

**Preliminary study 3**

From the results of preliminary study 2, we selected the words to include in the Scrambled-sentence task prioritizing those from Kay and Ross´ (2003) article, and those perceived to relate to the target words intermediately or highly. We also looked for similarity in the mean word scores between conditions.

Once the adapted Scrambled-sentence task was ready, we conducted a third preliminary study to evaluate and compare the perceived difficulty and time of completion of the task between the cooperative and competitive conditions. A total of 18 students from the National University of Cordoba completed the scrambled-sentence task for the cooperation (n = 9) or competition (n = 9) conditions and indicated the perceived difficulty in a scale ranging from 1 (very easy) to 10 (very difficult). We also measured the time of completion using a manual timer. The task was completed in paper-and-pencil, individually in the campus of the university. In both conditions the mode was to perceive the task as easy (Mo = 3). There neither were differences on perceived difficulty (n = 16; Cooperation M = 4, SE = .567; Competition M = 3.75, SE = .701; *t*(14) = -.277, 95% CI [-2.18, 1.68],*p* = .786), nor in time of completion (n = 18; Cooperation M = 394.89, DS = 60.32, SE = 20.10; Competition M = 393.44, DS = 82.34, SE = 27.44; *t*(16) = -0.042,95% CI [-73.57, 70.68],*p* = 0.967). A complete analytic dataset is available on osf.io/m9gh2.
